# Unusual Cysteine Content in V1 Region of gp120 From an Elite Suppressor That Produces Broadly Neutralizing Antibodies

**DOI:** 10.3389/fimmu.2019.01021

**Published:** 2019-05-15

**Authors:** Jennie M. Hutchinson, Kathryn A. Mesa, David L. Alexander, Bin Yu, Sara M. O'Rourke, Kay L. Limoli, Terri Wrin, Steven G. Deeks, Phillip W. Berman

**Affiliations:** ^1^Department of Biomolecular Engineering, University of California, Santa Cruz, Santa Cruz, CA, United States; ^2^Monogram Biosciences, South San Francisco, CA, United States; ^3^Department of Medicine, University of California, San Francisco, San Francisco, CA, United States

**Keywords:** HIV, vaccine, gp120, Env, disulfide, supressor, controller, provirus

## Abstract

Although it is now possible to produce recombinant HIV envelope glycoproteins (Envs) with epitopes recognized by the 5–6 major classes of broadly neutralizing antibodies (bNAbs), these have failed to consistently stimulate the formation of bNAbs in immunized animals or humans. In an effort to identify new immunogens better able to elicit bNAbs, we are studying Envs derived from rare individuals who possess bNAbs and are able to control their infection without the need for anti-retroviral drugs (elite supressors or ES), hypothesizing that in at least some people the antibodies may mediate durable virus control. Because virus evolution in people with the ES only phenotype was reported to be limited, we reasoned the Env proteins recovered from these individuals may more closely resemble the Envs that gave rise to bNAbs compared to the highly diverse viruses isolated from normal progressors. Using a phenotypic assay, we screened 25 controllers and identified two for more detailed investigation. In this study, we examined 20 clade B proviral sequences isolated from an African American woman, who had the rare bNAb/ES phenotype. Phylogenetic analysis of proviral envelope sequences demonstrated low genetic diversity. Envelope proteins were unusual in that most possessed two extra cysteines within an elongated V1 region. In this report, we examine the impact of the extra cysteines on the binding to bNAbs, virus infectivity, and sensitivity to neutralization. These data suggest structural motifs in V1 can affect infectivity, and that rare viruses may be prevented from developing escape.

## Introduction

Despite the widespread availability of anti-retroviral drugs, recent studies suggest that an HIV vaccine will still be required to control and eliminate the spread of this virus (1, 2). Over the last decade, evidence has accumulated from passive immunization studies to suggest that a vaccine that elicits broadly neutralizing antibodies (bNAbs) to the HIV envelope glycoprotein (Env), prior to infection, can provide protective immunity against HIV (3–11). Although considerable progress has been made in developing immunogens, such as properly folded trimeric Envs (12–19) that accurately replicate the antigenic structures of the 5–6 major epitopes recognized by bNAbs (20–22), none have been effective in eliciting protective neutralizing antibodies (23–28). Thus, while the immunogens developed to date possess the proper antigenic structure, the epitopes themselves appear to be poorly immunogenic, and we have not yet replicated the immunogenic structure required to elicit bNAbs.

A number of different strategies are being pursued to improve recombinant Env immunogenicity. One approach involves immunization with properly folded and glycosylated fragments of Env proteins (29–33) in hopes of selectively stimulating the formation of bNAbs, while precluding the possibility of stimulating antibodies to immunodominant non-neutralizing epitopes. A second approach to this problem has been to employ guided immunization strategies, using a prime-boost series that reconstruct the ontogeny of bNAb evolution (23, 24, 34–38). A third approach considers the possibility that Envs recovered from rare individuals with high levels of bNAbs, termed elite neutralizers (ENs), may be more effective in eliciting bNAbs than Envs from normal progressors that failed to produce bNAbs. However, the task of antigen selection from ENs is complicated by the fact that bNAbs are typically detected 2 years or more after infection (39, 40). By this time, Env sequences have diversified considerably and most circulating plasma viruses are enriched for neutralization resistant variants (41, 42). Thus, finding the precursor envelope sequences likely to have elicited bNAbs in an EN is a formidable challenge.

Here, we consider whether Env proteins closely resembling those that stimulated the formation of bNAbs can be recovered from rare individuals where virus evolution has been restricted or slowed, but still possess bNAbs. Previous studies have reported that virus evolution is considerably limited in individuals termed elite suppressors (ES) that are defined by their ability to limit virus replication without the need for antiretroviral drugs (43–52). Less than 1% of HIV-positive individuals possess the ES phenotype, meaning their viral load is less than detectable levels, i.e., <50 or <75 RNA copies/ml, for more than a year without anti-retroviral treatment (53). The ability to control viral load correlates with improved disease outcomes but the mechanism remains unclear. ESs exhibit heterogeneous immune responses but virus-specific CD8+ T cell responses appear to be a dominant feature (43, 54–58). ESs typically have viral populations with both limited genetic diversity and lower replication rates than normal progressors, including ENs (43–52). In addition, HIV in ESs may be constrained due to their fitness landscape, where mutational escape is limited due to the high fitness cost of mutations (43, 44, 59–64). Although most ESs fail to produce bNAbs, rare individuals possess bNAbs with neutralization breadth and exhibit the ES phenotype (bNAbs/ES phenotype). The restrictions on virus evolution in these individuals raised the possibility that Envs from individuals with the bNAb/ES phenotype may be more closely related to those that elicited the production of bNAbs than normal progressors with the EN phenotype. Therefore, we wondered if Env proteins from ESs that produce bNAbs might be enriched for unusual structural features, possibly related to epitope stabilization, antigen processing, or antigen presentation, that may have enhance immunogenicity leading to the formation of bNAbs. Here, we describe the recovery of 20 functional proviral sequences isolated from an individual who is an elite suppressor (viral load <75 HIV RNA copies/ml for over 4 years) and possesses bNAbs. We show that the majority of these sequences possess an unusual number of cysteines that may form an additional disulfide bond.

## Materials and Methods

### Clinical Specimens

Twenty-nine blinded archival plasma specimens (0.5–2.0 ml) from anti-retroviral therapy (ART) naïve men and women were obtained from the SCOPE study cohort (University of California, San Francisco, San Francisco, CA). Twenty-five of the specimens were from individuals previously identified as elite supressors (<75 HIV RNA copies/ml for 12+ months without ART). Four specimens were from normal progressors. Specimens were collected according to an Institutional Review Board (IRB) approved protocol for which study participants provided written consent and were seen at regular intervals. At each visit, subjects took a confidential questionnaire, were invited to participate in a medical exam for additional studies, and a blood sample was taken. After screening plasma for virus neutralizing antibodies (described below) an archival contemporaneous sample of non-viable PBMCs was obtained from an individual (designated as “EN3”) with the dual ES and broadly neutralizing antibody phenotype for sequence analysis.

Samples from a separate cohort, which included patient “EN1,” were collected by a physician under an Institutional Review Board (IRB) approved protocol from volunteers attending a regional center for recruitment in the San Francisco Bay Area. Inclusion criteria stipulated HIV-positive men and women 18–65 years of age, HIV ELISA or Western Blot positive for at least 1 year prior to screening, and who have never received anti-retroviral therapy (including post-exposure prophylaxis). An initial 10 mL of blood was collected into two EDTA tubes and plasma were aliquoted into cryovials and tested for bNAbs (described below). Four individuals with high titers of bNAbs were asked to participate in a 500 mL blood draw in a clinical setting. The blood was processed by a commercial laboratory using standard techniques and the PBMCs and plasma were separated, aliquoted, and cryopreserved under conditions that preserved plasma virus RNA and cell associated provirus DNA. The specimens were stored at −80°C until further analysis.

### Screen for Neutralization Breadth

Plasma samples were tested for virus neutralizing activity at Monogram Biosciences (South San Francisco, CA) in a standard pseudotype virus neutralization assay (PhenoSense®) using a panel of either 22 or 26 international isolates widely used in HIV vaccine research. Briefly, pseudotype viruses were prepared by cotransfecting HEK293 cells (American Type Culture Collection [ATCC], Manassas, VA) with an Env expression vector and an Env-deficient HIV-1 genomic vector carrying a luciferase reporter gene. Serial dilutions of MAbs or plasmas/sera were incubated with pseudotype viruses for 1 h prior to addition of U87 cells expressing CD4, CCR5, and/or CXCR4. The Z23 serum was used at an initial dilution of 1/100 and was included as an internal control in all experiments. Neutralization data are reported as 50% inhibitory dilutions (ID50s) for serum or plasma calculated from 5 point serum dilution curves. The virus controls included pseudoviruses prepared from the neutralization-sensitive HIV-1 isolate NL4-3 and the less neutralization-sensitive primary isolate JRCSF. The negative-control virus consisted of pseudotype viruses prepared from the envelope of the amphotropic murine leukemia virus (aMLV). HIV-1 neutralization titers were considered significant only if they were >3 times higher than the aMLV titers. The neutralization assays were performed according to good laboratory practice (GLP) and using protocols approved under Clinical Laboratory Improvement Amendments (CLIA). Each assay included acceptability criteria to ensure that interassay variation between ID50s, measured with reference standards, fell within 2.5-fold 95% of the time (65).

### Recovery of Env Sequences and Tropism Assay

An attempt was made to recover virus sequences from plasma and PBMCs from EN3. Due to the low copy number, the PhenoSense® assay system of Monogram Biosciences was unable to recover full-length functional clones of HIV envelope genes from plasma. However, full-length functional clones of HIV envelope genes were recovered from provirus DNA in PBMCs. A unique feature of Monogram Biosciences' assay system is a selection step for functional Envs that eliminates defective and noninfectious envelope sequences common in proviral specimens. Viral tropism was determined using the Trofile® DNA assay system from Monogram Biosciences. The DNA sequences of the resulting clones were determined by Sanger chain termination sequencing and analyzed for genetic clade using the Recombinant Identification Program (RIP) HIV clade assignment tool (66).

### Phylogenetic Analyses

The gp160 sequences were aligned in Geneious v5.6.7 (67) using the MUSCLE algorithm (68). The sequence of the JRCSF isolate of HIV was designated as the outgroup. The evolutionary history was inferred by using the Maximum Likelihood method based on the General Time Reversible model (69). The tree with the highest log likelihood (−8098.9704) is shown. The percentage of trees in which the associated taxa clustered together is shown next to the branches. Initial tree(s) for the heuristic search were obtained automatically by applying the Neighbor-Joining method to a matrix of pairwise distances estimated using the Maximum Composite Likelihood (MCL) approach. A discrete Gamma distribution was used to model evolutionary rate differences among sites [5 categories (+G, parameter = 0.3273)]. The tree is drawn to scale, with branch lengths measured in the number of substitutions per site. The analysis involved 32 proviral nucleotide sequences (20 from EN3, 11 from EN1, an elite neutralizer with normal progression, and JRCSF). All positions with <95% site coverage were eliminated. That is, fewer than 5% alignment gaps, missing data, and ambiguous bases were allowed at any position. There were a total of 2505 positions in the final dataset. Evolutionary analyses were conducted in MEGA6 (70).

### Expression of Recombinant gp120

Codon-optimized gene sequences were used for the expression of gp120. Synthetic gp120 genes (Invitrogen Inc., Waltham, MA) were inserted into an in-house plasmid expression vector (pCF1) containing a CMV promoter using standard techniques. PCR site-directed mutagenesis was performed to create variants of gp120 using a standard protocol for Gibson Assembly (New England Biolabs, Inc, Ipswich, MA). Recombinant plasmid sequences were confirmed by Sanger sequencing (University of California Sequencing Facility, Berkeley, CA). DNA was extracted and purified with a maxiprep kit (Qiagen, Redwood City, CA). For gp120 expression studies, envelope genes were transiently transfected into a HEK293 cell variant lacking the enzyme N-acetylglucosaminyltransferase I (HEK293S GnTI-, ATCC® CRL-3022TM) (29). The cells were transfected using polyethylenimine, grown in serum free cell culture medium (FreeStyle TM 293F; Invitrogen, Inc., Waltham, MA), and harvested after 5 days of growth. The gp120 proteins were expressed as fusion proteins that possessed an N-terminal flag epitope of 27 amino acids from herpes simplex virus 1 glycoprotein D (gD-1) as described previously (71, 72).

### Immunoblots

Growth conditioned cell culture supernatants with and without 25 mM DTT were analyzed for identity and molecular mass on 8–12% NuPage SDS-PAGE gel in MES running buffer and transferred to a PDVF membrane using standard protocol for iBlot (ThermoFisher Scientific, Waltham, MA). Membrane was blocked in 5% milk overnight on shaker at room temperature then washed 3 times with phosphate buffer saline with 0.01% Tween 20 (PBST; Sigma-Aldrich, St. Louis, MO) for 10 min. Membrane was probed with 5 μg/ml rabbit polyclonal anti-rgp120 antibody from previous immunization study (PB94) (71) in 5% milk for 2 h on shaker at room temperature, washed, then probed with 1:5,000 dilution of HRP-conjugated anti-rabbit secondary antibody (Jackson ImmunoResearch, West Grove, PA) in 5% milk for 2 h on shaker at room temperature and washed again. Antibody was detected using WesternBright reagents (Advansta, Menlo Park, CA) and visualized using an Innotech FluoChem2 system (Genetic Technologies Grover, MO).

### Antibody Binding Assays

A fluorescent immunoassay (FIA) was used to measure antibody binding. Briefly, 96 well plates (Greiner, Bio-One, USA) were coated with 60 μl per well of 2 μg/ml of mouse anti-gD antibody (34.1) in phosphate buffer saline (PBS) overnight. Plates were blocked by adding 100 μl per well of 1% bovine serum albumin (BSA) in PBS on a shaker for 2 h at room temperature. Plates were then washed four times with 100 μl per well of PBST. Sixty microliter of supernatant was added and incubated overnight at 4°C then washed again. Serial 1:3 dilutions of monoclonal human antibody in PBS were added to each well and incubated for 90 min on a rocker at room temperature then washed again. Plates were then probed with 100 μl per well of Alexa Fluor 488 goat anti-human IgG secondary antibody (ThermoFisher Scientific, Waltham, MA) at a 1:5,000 dilution in 1% BSA PBS for 90 min on a rocker at room temperature then washed again. Antibodies were detected by adding 50 μl per well of PBS and visualizing at 495 nm. FIA was performed in triplicate.

### Virus Neutralization Assays

Envelope gene sequences recovered from provirus clones were chemically synthesized *de novo*. Synthetic wild type (WT) non-codon-optimized gp160 gene sequences (Invitrogen Inc., Waltham, MA) were inserted into an in-house plasmid expression vector (pCF1) containing a CMV promoter using standard techniques. PCR site-directed mutagenesis was performed to create variants of gp160 using a standard protocol for Gibson Assembly (New England Biolabs, Inc, Ipswich, MA). Recombinant plasmid sequences were confirmed by Sanger sequencing (University of California Sequencing Facility, Berkeley, CA). DNA was extracted and purified with a maxiprep kit (Qiagen, Redwood City, CA). The plasmids were transferred to Monogram Biosciences (South San Francisco, CA) for testing in a pseudotype virus neutralization assay (PhenoSense®). The pseudotype viruses were tested for sensitivity and resistance to neutralization by antibodies in autologous contemporaneous plasma, as well as a panel of broadly neutralizing antibodies provided by the NIH AIDS Reagent Program, Polymun Scientific (Vienna, Austria), and Dr. Dennis Burton (Scripps Clinic and Research Institute, La Jolla, CA). The neutralizing antibody titer (IC50) for monoclonal antibodies is defined as the concentration of purified mAb (μg/L) that produces a 50% reduction in target cell infection.

## Results

### Identification of a Treatment-Naïve Elite Suppressor With Neutralization Breadth

Twenty-nine plasma specimens from ART-naïve individuals were obtained from the SCOPE Study cohort (University of California, San Francisco) and screened for neutralization breadth with a panel of 22 international virus isolates from five different genetic clades (Supplementary Table 1). Twenty-five of the plasma specimens were from individuals previously identified as elite supressors (ESs) and four specimens were from normal progressors. One specimen from a normal progressor (Z23) from a different cohort previously identified as possessing broad and potent neutralizing antibodies was used as a positive control. The panel included 5 viruses reported by Simek et al. (73) previously used to screen for elite neutralizers. Two specimens isolated from elite suppressors, EN2 and EN3, were effective in neutralizing 17 or 18 of the 22 viruses in the panel (respectively). Based on this result and the availability of clinical specimens, we selected one individual (EN3) for further study. EN3 is an African American woman who was 47 years old and HCV negative at the time of collection. Clinical data showed EN3 possessed < 75 HIV RNA copies/ml for over 4 years. Plasma from EN3 was tested against an expanded panel of viruses and was capable of neutralizing 69% of viruses tested from clades A, B, C, D, and AE, with some ID50 titers above 300 (Table 1).

**Table 1:** Expanded pseudovirus panel to define neutralization breadth in elite suppressor (EN3).

			**ID50 (1/dilution)**	
	**Virus**	**Clade**	**EN3 plasma**	**Positive control (Z23)**
Simek panel	94UG103	A	**439**	**112**
	92BR020	B	**268**	**226**
	93IN905	C	168	**253**
	M-C-026	C	84	**234**
	92TH021	AE	128	**157**
	JRCSF (Pos. Control)	B	**444**	**225**
	NL43 (Pos. Control)	B	**3,637**	**1,288**
	aMLV (Neg. Control)	N/A	69	< 100
Expanded panel	92RW008	A	**728**	**636**
	92RW020	A	**419**	**414**
	M-A-002	A	**102**	**198**
	M-A-009	A	**378**	**308**
	6535.3	B	26	**359**
	Bal	B	**145**	**726**
	MN	B	**790**	**12797**
	PV04	B	**344**	**319**
	REJO	B	**294**	**397**
	TRO.11	B	**660**	**306**
	98IN022	C	51	**207**
	M-C-020	C	40	**103**
	TV1	C	36	< 100
	92UG005	D	**247**	**396**
	94UG114	D	**129**	**162**
	M-D-006	D	**412**	**441**
	M-D-009	D	48	**270**
	3017_E10_113035_081	AE	**1,763**	**372**
	3178_E10_142902_080	AE	**298**	**493**
	CM244/A244	AE	**68**	**526**
	TH023	AE	**93**	**262**
	JRCSF (Pos. Control)	B	**208**	**217**
	NL43 (Pos. Control)	B	**2,734**	**1,137**
	aMLV (Neg. Control)	N/A	< 20	< 100

*EN3, an elite suppressor, was screened using Monogram Biosciences' PhenoSense® assay for neutralization breadth against a panel of 26 viruses. Each assay included acceptability criteria to ensure that interassay variation between ID50s, measured with reference standards, fell within 2.5-fold 95% of the time. The neutralizing antibody titer (ID50) is defined as the reciprocal of the plasma dilution that produces a 50% inhibition in target cell infection. Values in bold represent neutralization titers that are at least three times greater than those observed against the negative control (aMLV). The clade B NL4-3 and JRCSF viruses were included as CXCR4- and CCR5-dependent positive controls, respectively. Z23 is a reference serum possessing broadly neutralizing antibodies*.

### Phylogenetic Analysis of an Elite Suppressor and a Normal Progressor Illustrates Significant Difference in Genetic Variation

**Figure 1:**
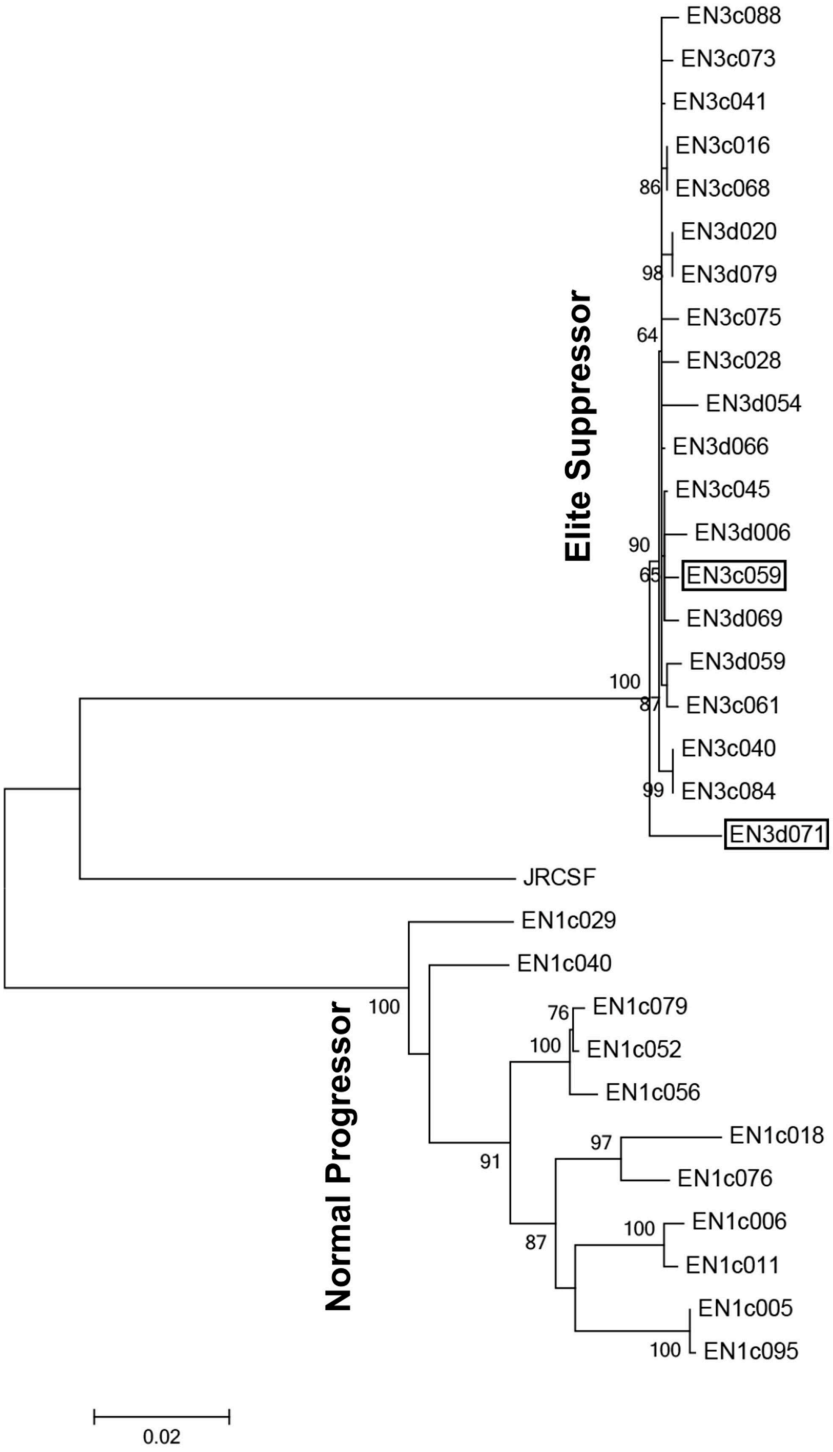
Phylogenetic analysis of proviral sequences isolated from an elite suppressor (EN3) and a normal progressor (EN1) that both possess bNAbs. Proviral sequences were recovered from two individuals (EN1 and EN3) using the Monogram Phenosene® assay system. Evolutionary analyses were conducted in MEGA6 using a Maximum Likelihood method with 1000 bootstrap replicates and a discrete Gamma distribution to model evolutionary rate. The tree is drawn to scale, with branch lengths measured in the number of substitutions per site. Sequences used in mutagenesis studies, EN3d071 and EN3c059, are shown with a black rectangle.

Phylogenetic analysis compared proviral sequences isolated from the elite suppressor, EN3, and a normal progressor, EN1, which both possessed broadly neutralizing antibodies. These were analyzed with MEGA6 to generate a maximum likelihood tree using the General Time Reversible (70) model with 1,000 bootstrap replicates. EN3 sequences had less intra-patient genetic variation compared to proviral sequences from EN1, who showed similar neutralization breadth (Figure 1). EN3 had 126 (4.8%) sites with polymorphisms whereas EN1 had 396 (14.8%) sites with polymorphisms. The result conformed to previous observations, reporting less genetic variation and slower viral evolution in an elite suppressor (EN3) compared to a normal progressor (EN1).

### Characterization of Proviral gp160 Sequences Isolated From EN3

**Figure 2:**
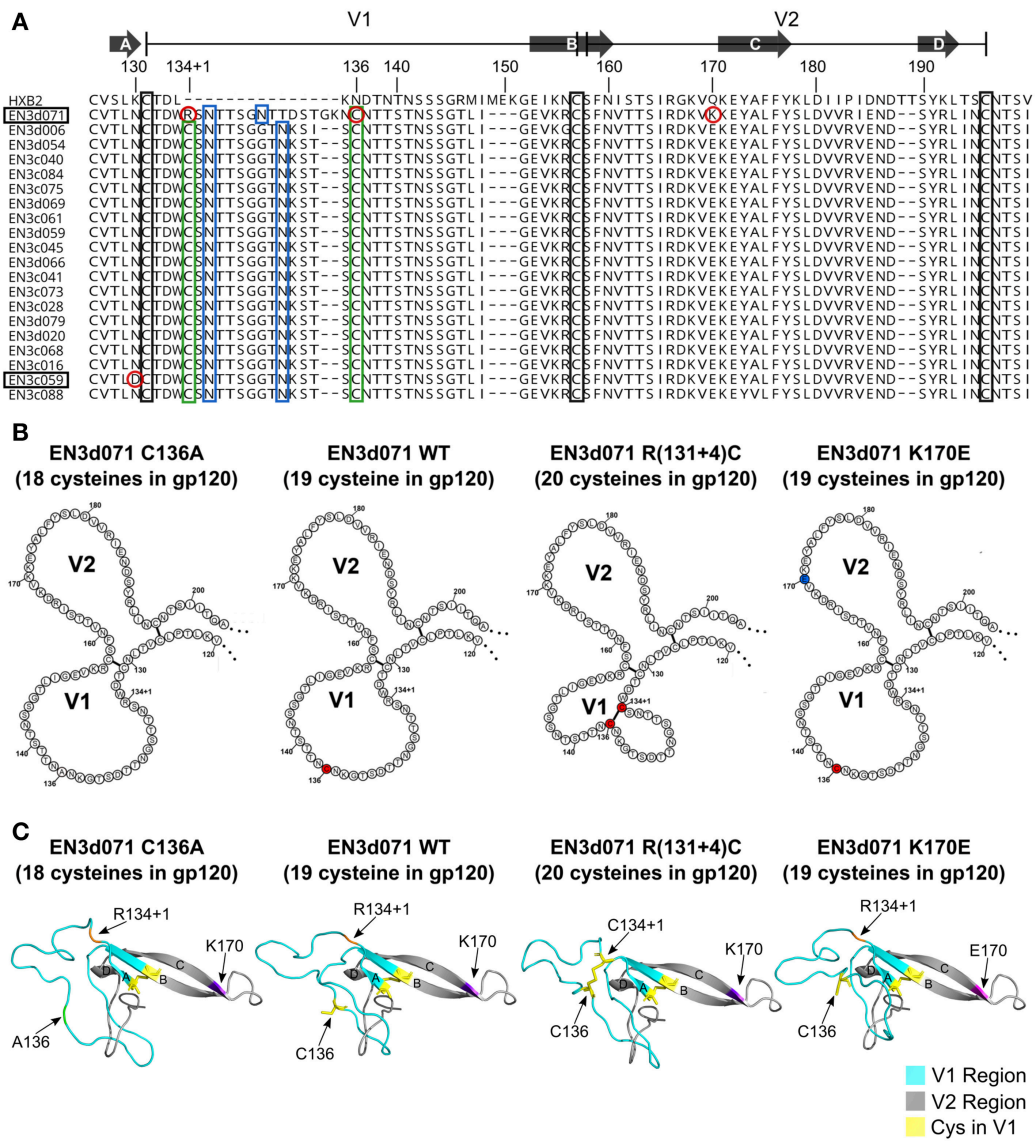
V1/V2 regions of proviral sequences isolated from an elite suppressor (EN3). **(A)** Alignment of functional proviral sequences isolated from an elite suppressor (EN3). V1 region is defined as amino acids 131–157 and V2 region is defined as amino acids 158–196 using sequence numbering relative to HXB2 reference standard. Arrows above the alignment show the location of four beta strands described in McLellan et al. (13). Black rectangles show the location of canonical cysteines in the V1/V2 region. Green rectangles show aberrant cysteines in functional sequences isolated from EN3. Blue rectangles show potential N-linked glycosylation sites that are part of the 13 or 15 amino acid insertion in V1. Residues circled in red were targeted in mutagenesis experiments. **(B)** Primary structure of EN3d071 wild type (WT) and mutants. Single point mutations of EN3d07 WT were introduced to create three different mutants of Env (EN3d071 C136A with no extra cysteines in V1, EN3d071 R(134+1)C with two extra cysteines in V1, and K170E with one extra cysteine in V1). Non-canonical cysteines are shown in red. K170E mutation is shown in blue. **(C)** Homology models of gp120s EN3d071 wild type (WT) and mutants. Homology models of EN3d071 C136A (18 cysteines), EN3d071 WT (19 cysteines), EN3d071 R(134+1)C (20 cysteines), and EN3d071 K170E (19 cysteines) were built using Modeler v9.21 with 5fykG template. V1 regions are shown in cyan. V2 regions are shown in gray. Cysteines located in the V1 region are shown in yellow. Polymorphisms are labeled with A136 shown in green, R(134+1) shown in orange, K170 shown in purple, and E170 shown in magenta.

The physical characteristics of the gp160 sequences were analyzed for amino acid length, the number of cysteines, and location of potential N-linked glycosylation sites in each region (Table 2). Sequences were compared to the Los Alamos National Laboratory database (74) filtered for clade B subtype envelope sequences, with one sequence per individual. EN3 had a significantly longer V1 region (37–39 amino acids; Kruskal-Wallis test, *p* < 0.0001) and rare cysteines in the V1 hypervariable region (3–4 cysteines; Kruskal-Wallis test, *p* < 0.0001) compared to the dataset from LANL (average V1 length is 27 amino acids; average number of cysteines in V1 is 2). When the recovered sequences were aligned (Figure 2), all of the sequences with the exception of clone d071 possessed two additional N-linked glcosylation sites and two extra cysteines (Cys) in the V1 region. The extra Cys residues occurred at fixed locations (C134+1 and C136 using HXB2 numbering), resulting in a total of 20 Cys in the gp120 fragment compared to the normal 18 Cys typically found in the major (M) class of HIV envelope sequences. The EN3d071 Env possessed a Cys at position 134+1, like all of the other viruses from this individual, but had the polymorphism C136R, which is adjacent to a 15 amino acid insertion between HXB2 positions 134 and 135 (Figure 2).

**Table 2:** Physical characteristics of 20 functional proviral sequences isolated from EN3.

**Region**	**HXB2 numbering**	**Length (aa)**		**PNGS**		**Cysteines**	
		**Mean ± SD**	**Range**	**Mean ± SD**	**Range**	**Mean ± SD**	**Range**
Signal	1–30	29.0 ± 0.0	29–29	0.0 ± 0.0	0 – 0	1.0 ± 0.0	1–1
C1	31–130	100.0 ± 0.0	100–100	1.9 ± 0.2	1–2	4.0 ± 0.2	4–5
V1	131–157	37.1 ± 0.4^*^	**37**–39^*^	4.0 ± 0.0	4–4	4.0 ± 0.2^*^	**3**–4^*^
V2	158–196	37.0 ± 0.0	37–37	2.0 ± 0.0	2–2	1.0 ± 0.0	1–1
C2	197–295	99.0 ± 0.0	99–99	6.0 ± 0.0	6–6	5.0 ± 0.0	5–5
V3	296–331	35.0 ± 0.0	35–35	1.0 ± 0.0	1–1	2.0 ± 0.0	2–2
C3	332–384	53.0 ± 0.2	52–53	3.0 ± 0.0	3–3	1.0 ± 0.0	1–1
V4	385–418	38.0 ± 0.0	38–38	5.0 ± 0.3	4–6	2.0 ± 0.0	2–2
C4	419–459	41.0 ± 0.0	41–41	1.0 ± 0.0	1–1	1.0 ± 0.0	1–1
V5	460–469	11.0 ± 0.0	11–11	1.0 ± 0.0	1–1	0.0 ± 0.0	0–0
C5	470–511	42.0 ± 0.0	42–42	0.0 ± 0.0	0–0	0.0 ± 0.0	0–0
gp41 extracellular	512–678	167.0 ± 0.0	167–167	3.0 ± 0.0	3–3	2.0 ± 0.0	2–2
gp41 transmembrane	679–699	21.0 ± 0.0	21–21	0.0 ± 0.0	0–0	0.0 ± 0.0	0–0
gp41 cytoplasmic tail	700–856	157.0 ± 0.0	157–157	1.0 ± 0.0	1–1	1.0 ± 0.0	1–1
gp120	31–511	493.1 ± 0.2	493–494	24.9 ± 0.4	24–26	20.0 ± 0.3	19–21
gp41	512–856	345.0 ± 0.0	345–345	4.0 ± 0.0	4–4	3.0 ± 0.0	3–3
gp160	31–856	838.0 ± 0.2	838–839	28.9 ± 0.4	28–30	23.0 ± 0.3	22–24

The number of amino acids, potential N-linked glycosylations sites, and cysteines in each region were compared to clade B sequences (Los Alamos National Laboratory HIV database). As indicated in bold, EN3 had a significantly longer V1 region (37–39 amino acids) and had rare cysteines in the V1 hypervariable region (3–4 cysteines) compared to the dataset from LANL (average V1 length is 27 amino acids; average number of cysteines in V1 is 2).

* *p < 0.0001 for the Kruskal-Wallis test comparing features to the Los Alamos National Laboratory dataset*.

### Sensitivity to Neutralization by Polyclonal Antibodies in Autologous Plasma and Broadly Neutralizing Monoclonal Antibodies (bN-mAbs)

Pseudotype viruses representing the 20 envelope clones from EN3 were constructed and tested for sensitivity to neutralization by contemporaneous autologous plasma and by a panel of prototypic bN-mAbs (Table 3). We observed that all viruses were sensitive to neutralization by autologous plasma (range 1:322–1:53,788) with a mean neutralization titer of 1:3,376. However, one clone, EN3c059, was exceedingly sensitive to neutralization by autologous plasma with a neutralization titer of 1:53,788. We observed that this clone was also particularly sensitive to neutralization by the positive control Z23.

**Table 3:** EN3 pseudotype virus neutralization titers from autologous sera and a panel of bN-Abs.

**Proviral clones**	**ID50 (1/dil'n)**	**IC50 (ug/ml)**								**ID50 (1/dil'n)**
	**Autologous sera**	**PG9**	**PG16**	**PGT145**	**PGT121**	**PGT128**	**VRC01**	**4E10**	**35022**	**Positive control (Z23)**
EN3c016	**525**	>25	>25	**3.5588**	>25	**0.0255**	**1.7871**	>25	>25	**296**
EN3c028	**534**	>25	>25	**3.4674**	>25	**0.0277**	**1.6674**	**17.3799**	>25	**400**
EN3c040	**322**	>25	>25	**5.0631**	>25	**0.0277**	>25	**13.9435**	>25	**212**
EN3c041	**500**	>25	>25	**3.1332**	>25	**0.0219**	**1.3681**	**23.1582**	>25	**303**
EN3c045	**881**	>25	>25	**0.9347**	>25	**0.0158**	**0.5813**	**2.627**	**3.585**	**1026**
EN3c059	**53788**	>25	>25	>25	>25	**0.1172**	**21.4415**	**0.5471**	>25	**90980**
EN3c061	**1151**	>25	>25	**1.2681**	>25	**0.0143**	**0.6382**	**2.6453**	**0.0058**	**1171**
EN3c068	**479**	>25	>25	**2.5685**	>25	**0.024**	**2.0384**	>25	>25	**446**
EN3c073	**600**	>25	>25	**6.194**	>25	**0.0257**	**1.3944**	**12.2846**	>25	**503**
EN3c075	**392**	>25	>25	**3.1324**	>25	**0.0289**	**2.005**	>25	>25	**335**
EN3c084	**426**	>25	>25	**4.7513**	>25	**0.0327**	>25	**11.5505**	>25	**230**
EN3c088	**513**	>25	>25	**6.7844**	>25	**0.0315**	**1.3092**	**15.1361**	>25	**393**
EN3d006	**509**	>25	>25	>25	>25	**0.0182**	**1.0039**	**22.4744**	>25	**437**
EN3d020	**574**	>25	>25	**3.2216**	>25	**0.0313**	**2.0042**	**16.9443**	>25	**336**
EN3d054	**1647**	>25	>25	**1.248**	>25	**0.0473**	**0.9561**	**3.4162**	>25	**611**
EN3d059	**1787**	>25	>25	**13.606**	>25	**0.0241**	**2.8993**	**11.2629**	**7.5214**	**1598**
EN3d066	**545**	>25	>25	**2.0665**	>25	**0.0186**	**0.3923**	>25	>25	**309**
EN3d069	**949**	>25	>25	**1.5781**	>25	**0.0182**	**0.8521**	**2.8472**	**23.4584**	**1152**
EN3d071	**886**	**3.5967**	**0.3081**	**0.1229**	>25	**0.0095**	**7.4507**	>25	**0.0038**	**323**
EN3d079	**510**	>25	>25	**2.8251**	>25	**0.0277**	**2.2708**	**9.4933**	>25	**317**

*Pseudotype viruses for each EN3 sequence were tested against autologous sera and a panel of bn-mAbs with Monogram Biosciences' PhenoSense® assay. Each assay included acceptability criteria to ensure that interassay variation between ID50s, measured with reference standards, fell within 2.5-fold 95% of the time. The neutralizing antibody titer (ID50) for autologous sera and Z23 (a reference serum possessing broadly neutralizing antibodies) is defined as the reciprocal of the plasma dilution that produces a 50% inhibition in target cell infection. The neutralizing antibody titer (IC50) for monoclonal antibodies is defined as the concentration of purified mAb (μg/ml) that produces a 50% reduction in target cell infection. Values in bold indicated neutralization sensitivity defined as ID50 values that are at least three times greater than those observed against the negative control (aMLV) or IC50 values that are < 25 μg/ml. EN3c059 and EN3d071 were selected for further study and are shown in gray*.

When we examined the sensitivity of the EN3 viruses to prototypic bN-mAbs, we found that all but clone EN3d071 were resistant to neutralization by the PG9 and PG16 broadly neutralizing monoclonal antibodies (bN-mAbs) known to bind to glycan-dependent epitopes in the V1/V2 domain (13). When we examined the sensitivity of these Envs to bN-mAbs that recognized glycan-dependent epitopes at the base of the V3 domain (14), we found that all of the clones were sensitive to neutralization by the PGT128 bN-mAb but all of the clones were resistant to neutralization by the PGT121 bN-mAb. We found that 18 of 20 clones were sensitive to neutralization by the VRC01 bN-mAb specific for the CD4-binding site, and the PGT145 bN-mAb that recognizes a trimer-specific epitope involving the V1/V2 domain. In addition, 15 of 20 clones were sensitive to neutralization by the 4E10 bN-mAb specific for the membrane proximal external domain (MPER). Finally, 5 of 20 clones were sensitive to the 35022 bN-mAb specific to the gp120 and gp41 interface.

### Effect of Extra Cysteine Residues and Polymorphisms at Positions 130 or 170 on Sensitivity to Neutralization by bN-mAbs

Because the EN3d071 clone was the only envelope with 19 Cys residues and the only virus that was sensitive to neutralization by the PG9 and PG16 bN-mAbs, we carried out mutagenesis studies to examine the effect of 18, 19, and 20 Cys residues on virus infectivity and sensitivity to neutralization. In addition to lacking C(134+1), EN3d071 is the only sequence that had a lysine (K) at position 170 where the other 19 sequences had a glutamic acid (E) at this position (Figure 2). Previous studies have suggested that K170 can be important for PG9 binding (75). To examine potential effects of the aberrant V1 cysteines and K170, we used site-directed mutagenesis to create three variants of EN3d071 gp160. These included: (1) the EN3d071 C136A variant with 18 cysteines in gp120, (2) the EN3d071 R(134+1)C with 20 cysteines in gp120, and (3) the EN3d071 K170E variant with 19 cysteines in gp120 (Figure 2). The wild type and mutated gp160 genes were then used to create pseudoviruses and tested for sensitivity to neutralization by a panel of bN-mAbs, including PG9, and autologous sera.

When expressed as pseudoviruses, we found that the wild type (WT) EN3d071 Env (19 Cys in gp120) and the EN3d071 C136A variant (18 Cys in gp120) retained infectivity and both possessed the CCR5 chemokine receptor (R5) tropic phenotype (Table 4). In contrast, the R(134+1)C variant (20 Cys in gp120) that contained two non-canonnical cysteines was non-infectious. When the 19 Cys variant possessing the K170E variant was examined, we observed that it had reduced infectivity but still retained the R5 receptor phenotype.

**Table 4:** Infectivity of wild type and mutant EN3d071 pseudoviruses.

**Rank to relative light units (RLU) Key**					
**Proviral clones**	**Number of Cys in gp160**	**Tropism**	**R5 infectivity (Rank)**	**X4 infectivity (Rank)**	0 Rank < 5,000 RLU (no infectivity detected)
					0.5 Rank < 5,000 RLU (infectivity detected)
EN3d071 WT	22	R5	2	0	1 Rank = 5,001 to 15,000 RLU
EN3d071 R(134+1)C^*^	23	N/A	0	0	2 Rank = 15,001 to 150,000 RLU
EN3d071 C136A	21	R5	2	0	3 Rank = 150,001 to 1,000,000 RLU
EN3d071 K170E	22	R5	0.5	0	4 Rank = >1,000,000 RLU

Viral tropism was determined using the Trofile® DNA assay system from Monogram Biosciences. CD4+/U87 cells expressing CXCR4 or CCR5 coreceptor were inoculated with pseudoviruses. Infected cells produced luciferase. Tropism was determined by the relative light units (RLU) in lysed cells 72 h after inoculation.

* *Infectivity too low for use in the neutralization assay*.

We next examined the sensitivity to neutralization by autologous plasma, and the panel of bN-mAbs directed to different epitopes (Table 5). The loss of the cysteine in EN3d071 C136A may have increased resistance to neutralization by VRC01 but the difference in IC50 values is within the margin of error. The K170E mutation caused resistance to neutralization by PG9 and PG16 and surprisingly increased sensitivity to 4E10. Neutralization sensitivity to the PGT145, PGT128, 35022 bN-mAbs, and autologous plasma was preserved in all testable variants.

**Table 5:** EN3 wild type and mutant pseudoviruses sensitivity to neutralization by bN-Abs and autologous sera.

**Proviral clone**	**Mutation**	**Number of Cys in gp160**	**ID50 (1/dil'n)**	**IC50 (μg/ml)**								**ID50 (1/dil'n)**
			**Autologous sera**	**PG9**	**PG16**	**PGT145**	**PGT121**	**PGT128**	**VRC01**	**4E10**	**35,022**	**Z23**
EN3d071	None	22	**995**	**8.8474**	**0.1002**	**0.1687**	>25	**0.0071**	**21.3912**	>25	**0.0019**	**229**
	R(134+1)C^*^	23	NA	NA	NA	NA	NA	NA	NA	NA	NA	NA
	C136A	21	**754**	**10.9931**	**0.2234**	**0.2041**	>25	**0.0070**	>25	>25	**0.0030**	154
	K170E	22	**1,024**	>25	>25	**0.5077**	>25	**0.0041**	**11.0866**	**4.3028**	**0.0035**	**378**
EN3c059	None	23	**64,097**	>25	>25	>25	>25	**0.0983**	**20.385**	**0.1318**	**8.5338**	**55,331**
	D130N	23	**57,182**	>25	>25	**21.2259**	>25	**0.2095**	**23.6354**	**0.16**	**21.5527**	**56,567**

The neutralization sensitivity of EN3d071 wild type (WT) was compared to three variants with single point mutations. Pseudotype viruses for each EN3 sequence were tested against autologous sera and a panel of bn-mAbs with Monogram Biosciences' PhenoSense® assay. Each assay included acceptability criteria to ensure that interassay variation between measured with reference standards, fell within 2.5-fold 95% of the time. The neutralizing antibody titer (ID50) for autologous sera and Z23 (a reference serum possessing broadly neutralizing antibodies) is defined as the reciprocal of the plasma dilution that produces a 50% inhibition in target cell infection. The neutralizing antibody titer (IC50) for monoclonal antibodies is defined as the concentration of purified mAb (μg/ml) that produces a 50% reduction in target cell infection. Values in bold indicated neutralization sensitivity defined as ID50 values that are at least three times greater than those observed against the negative control (aMLV) or IC50 values that are < 25 μg/ml.

* *Infectivity too low for use in the neutralization assay*.

The EN3c059 clone was highly sensitive to neutralization by contemporaneous autologous sera (ID50 above 53000) and differed by only 3 point mutations, E32Q, N130D, and A533V from the EN3 consensus sequence. We wondered if the N130D polymorphism, which eliminates a potential N-linked glycosylation site, could account for the increased neutralization sensitivity of this variant. We used site-directed mutagenesis to express EN3c059 WT and a variant with the D130N mutation (EN3c059 D130N) and tested for sensitivity to neutralization by a panel of bN-mAbs and autologous sera. Surprisingly, we found that the D130N polymorphism did not affect sensitivity to neutralization by contemporaneous autologous sera (Table 5).

### Effect of Extra Cysteine Residues and K170E on bN-mAb Binding to gp120

**Figure 3:**
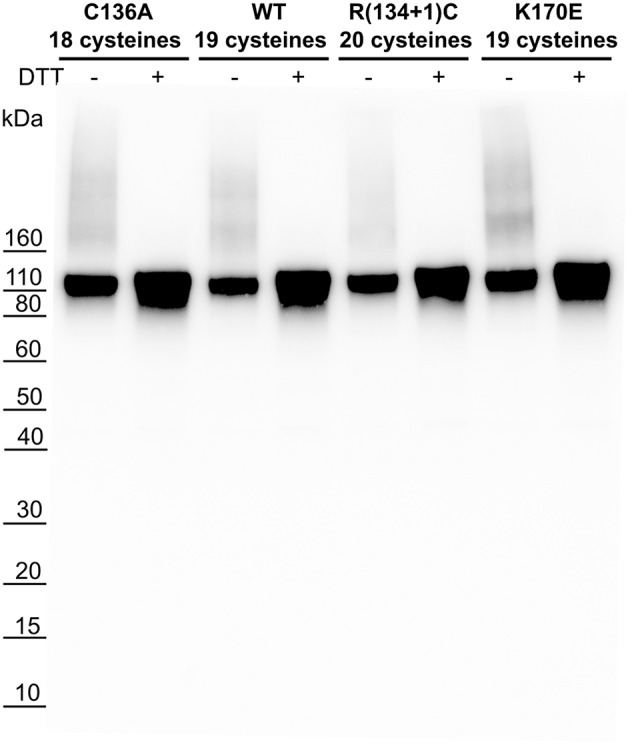
HEK293 GnTI- expression of recombinant gp120 EN3d071 WT and mutants. Recombinant gp120 sequences EN3d071 C136A (18 cysteines), EN3d071 WT (19 cysteines), EN3d071 R(134+1)C (20 cysteines), and EN3d071 K170E (19 cysteines) were transiently transfected in HEK293 GnTI- cells. HEK293 GnTI- cells lack N-acetylglucosaminyltransferase 1, disrupting the glycosylation pathway and resulting in Man5 glycans which have a smaller molecular weight. Supernatant with and without 25 mM DTT was run on SDS-PAGE gel, transferred to a PDVF membrane, and probed with purified rabbit polyclonal anti-gp120 antibodies (PB94) from a previous immunization study. EN3d071 WT and mutants showed similar expression levels with a small amount of aggregation detected in unreduced samples.

Additional studies were carried out to characterize bN-mAb binding to recombinant gp120s derived from EN3 envelope proteins. Because multiple bN-mAbs recognize epitopes that are dependent on mannose-5 (Man5) for binding, the Envs were expressed in HEK293 cells lacking N-acteylglucosaminyltransferase 1 (HEK293S GnTI- cells). The absence of N-acetylgucosaminyltransferase 1 disrupts the glycosylation pathway, resulting in the production of monomeric gp120s that contain a high mannose (Man5) glycan found on native HIV virions. The recombinant proteins predominately migrated as monomeric proteins and were not degraded by proteolysis often observed for Envs from clade B viruses (Figure 3). The EN3d071 R(134+1)C variant expressed well even though the pseudotype viruses made with this envelope were not infectious.

**Figure 4:**
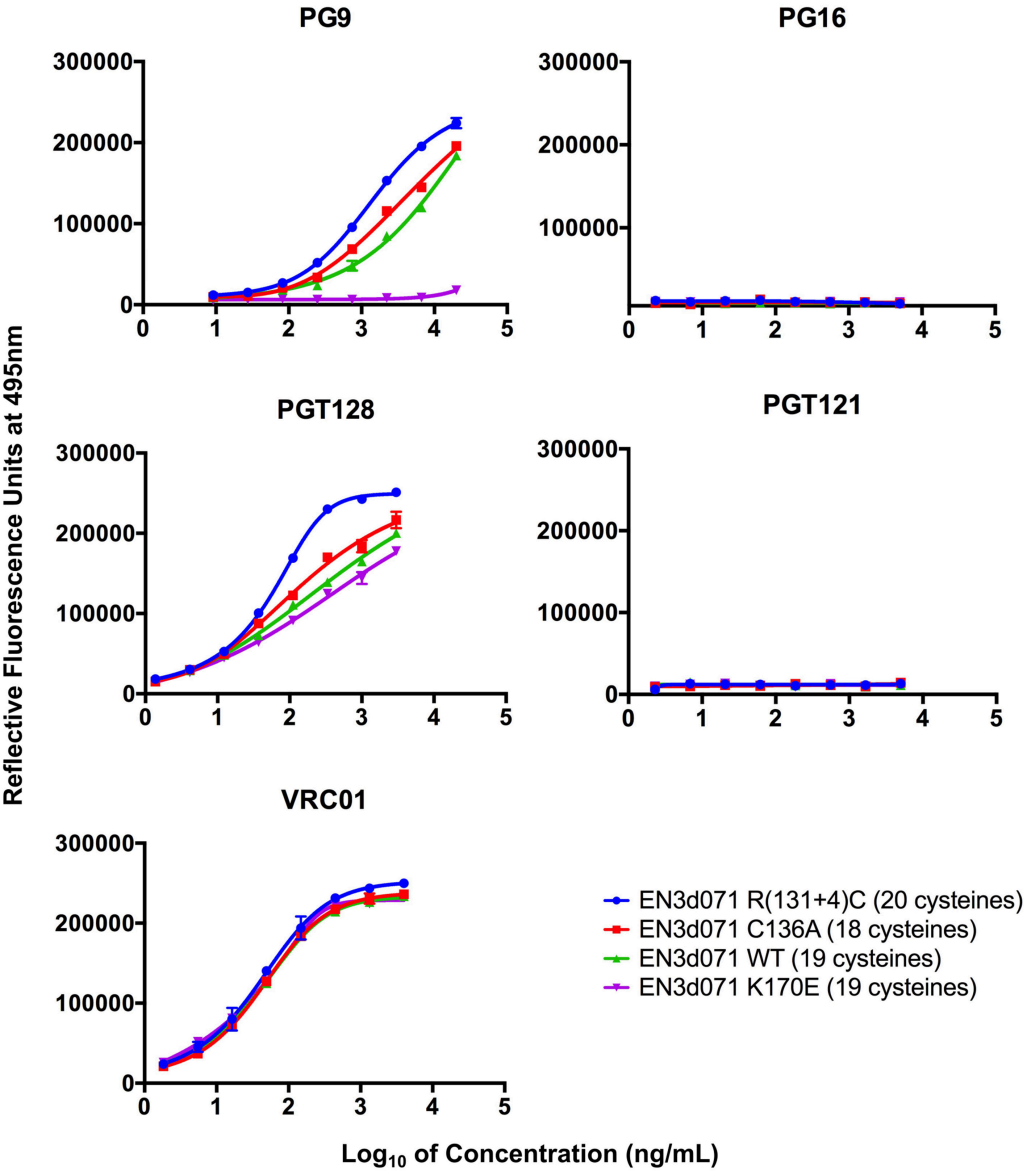
bN-mAb binding to recombinant gp120 EN3d071 WT and mutants by fluorescent immunoassay. Recombinant gp120 sequences EN3d071 C136A (18 cysteines), EN3d071 WT (19 cysteines), EN3d071 R(134+1)C (20 cysteines), and EN3d071 K170E (19 cysteines) were produced with an N-terminal gD tag and captured onto microtiter plates with a mouse monoclonal anti-gD antibody. The captured proteins were then incubated with a panel of broadly neutralizing monoclonal antibodies that included PG9, PG121, PGT128, and VRC01. After washing, the wells were incubated with Alexa Fluor 488 labeled goat anti-human IgG (ThermoFisher Scientific, Waltham, MA) and washed as described in Materials and Methods.

A fluorescence immunoassay (FIA) was used to characterize the binding of a panel of bN-mAbs to immunoaffinity purified variants of the EN3d071 Env protein. We found that the envelopes with 18, 19, and 20 cysteines all bound to PG9, PGT128, and VRC01 and did not bind to PGT121 (Figure 4). They also did not bind to PG16, which was expected because the gp120s were monomeric and lacked the hybrid complex glycans required for PG16 binding (13). Thus, all three proteins possessed the epitopes recognized by the PG9, PGT128, and VRC01 bN-mAbs but lacked the epitopes recognized by the PG16 and PGT121 bN-mAbs. EN3 K170E had a similar binding profile but did not bind to PG9. This provides additional evidence that the inability of PG9 to neutralize 19 of the 20 Envs recovered from EN3 appear to be attributable the E170K polymorphism in the V2 region rather than the extra pair of Cys residues in the V1 region.

## Discussion

In this paper, we have analyzed proviral Env sequences from a rare elite suppressor (EN3) with the bNAb/ES phenotype. As reported in previous studies (43–52), we found that proviral sequences from EN3 were highly homogeneous compared to proviral sequences recovered from a normal progressor. This result supports the idea that virus evolution is slower in ENs due to a reduced rate of virus replication. Because of this slow rate of virus evolution, we reasoned that it might be easier to recover Envs more closely related to those that elicited bNAb from individuals with the bNAb/ES phenotype than from normal progressors that exhibit much higher levels of proviral Env sequence diversity. Moreover, we postulated that analysis of sequences from individuals with bNAb/ES phenotype might provide clues regarding structural features that enhance for the formation of bNAbs compared to Envs from normal progressors that exhibit higher levels of virus diversification. The possibility that HIV has evolved structural features to modulate the immunogenicity of epitopes recognized by bNAbs is well supported by the unusually high degree of N-linked glycosylation resulting in a “glycan shield” (42), indels (insertions and deletions) in variable regions (76), and the observation that cleavage sites for antigen processing enzymes occur in close proximity to epitopes recognized by bNAbs (77).

In the present study, we found that Envs sequences recovered from EN3 were highly homogenous (99.1% pairwise identity) and 19 of 20 sequences displayed an unusual insertion in the V1 region that added a pair of extra cysteine residues, which may form an extra disulfide bond, and two additional N-linked glycosylation sites. The V1 region has long been known to be highly immunogenic and has previously been termed the “global regulator” of neutralization sensitivity (76). It is the first target of autologous neutralizing antibodies (20, 78–80). Evolution of glycosylation sites in the V1 region is thought to be a key factor in driving the evolution of bNAbs that normally occurs 2 years or more post infection (42, 78, 81–84). Additionally, the V1 region is located at the apex of the virus spike and is in close proximity to several epitopes recognized by bNAbs (13, 73, 85). Based on these observations, the three changes we have documented in the V1 region are all features of the type expected to affect either the antigenic structure or the immunogenicity of the Env protein.

In principle, 19 of 20 Envs from this individual could form 10 disulfide bridges compared to the 9 disulfide bridges found in most gp120 sequences. HIV-1 gp120 has 18 conserved cysteines that form 9 disulfide bridges present in all published crystal structures. Approximately 7% of clade B viruses from newly infected individuals also have 20 cysteines (86) and 5.5% of clade B viruses have 2 extra cysteines in the variable 1 (V1) region (87). The EN3d071 sequence was unusual in that it possessed only 19 Cys and therefore contained an unpaired Cys that could only form 9 disulfide bridges. Free Cys residues are unusual in most secreted proteins and often lead to the formation of dimers or aggregates. The prevalence of these extra cysteines raised the possibility that EN3 envelope proteins might provide a functional advantage perhaps related to enhanced virus infectivity, neutralization escape, or increased immunogenicity. Additionally, they provided for the possibility of rearrangements of disulfide structures.

Additional N-linked glycosylation sites and insertions in the V1/V2 domain are well documented immune escape mechanisms known to prevent the binding of bNAbs (42, 78, 81–84). Surprisingly, neither the extra glycosylation sites, the 13 amino acid insertion, nor the extra cysteines in the V1 region affected binding or sensitivity to neutralization by bN-mAbs PG9, PG16, PGT121, PGT128, PGT130, 35022, or 4E10. Therefore these changes may have evolved to serve another function, perhaps related to virus infectivity or stability. When this possibility was considered, it was interesting that the R(134+1)C variant of EN3d071, possessing 20 Cys residues, had little or no infectivity compared to the WT Env that possessed 19 Cys. All other clones possessing 20 Cys and the C(134+1) polymorphism were infectious, indicating epistatic mutations are required to compensate for the presence of the additional Cys. Similarly, the K170E variant of EN3d071 had reduced infectivity, despite the presence of E170 in all other clones. Thus, epistatic mutations are also required to preserve infectivity for the K170E polymorphism. Two of the four point mutations altering amino acids to match the consensus sequence reduced infectivity, perhaps indicating the fitness landscape is restricted.

Although we did observe a mutation that improved sensitivity to neutralization by PG9 binding, this mutation occurred in only one virus clone and was distinct from the insertion in the V1 region found in the 19 other viruses. The glutamic acid (E) at position 170 accounts for the resistance to neutralization by the PG9 bN-mAb in all of the viruses except EN3d071 that possesses a lysine (Lys) at this position. Previous studies reported that the K168E, K169E, and K171E polymorphisms disrupt PG9 binding and cause resistance to neutralization by PG9. This is likely due to the change in charge on the “C” beta strand in the V2 region, which is part of the PG9 epitope (13). It is consistent that K170E, located on the “C” beta strand, also disrupts binding and confers resistance to neutralization by PG9.

Based on these studies, the possibility remains that the insertion in the V1 region, the extra pair of cysteines, and the extra glycosylation sites may have contributed to the formation of bNAbs in EN3. It is important to note that the gp160 sequences studied here are a sample of the virus population taken at a single time point and may not include the actual viral sequences that stimulated the production of the bN-mAbs in contemporaneous plasma. However, variation within the 20 proviral sequences was minimal, supporting previous reports suggesting that viral evolution may be slower in this elite suppressors (45–54). These sequences may be more similar to their bN-mAb-inducing predecessor than viruses recovered from normal progressors, and thus better vaccine candidates. The envelopes described in this paper provide the basis for future immunization studies where the immunologic potential of individuals possessing the rare bNAb/ES phenotype can be examined in greater detail. To this end, efforts are in progress to create trimeric gp140s and DNA vectors as well as the monomeric gp120s described in this paper to further define the immunogenic properties of these Env proteins.

## Data Availability

This manuscript contains previously unpublished data. The name of the repository and accession number are not available.

## Author Contributions

JH, KM, and PB contributed to study design. JH and KM contributed to construct design and phylogenetic analysis. JH performed sequence alignments, protein expression and binding assays. DA and BY assisted in protein production. KL and TW performed neutralization assays. SD provided patient samples. All authors contributed to manuscript revision.

### Conflict of Interest Statement

The authors declare that the research was conducted in the absence of any commercial or financial relationships that could be construed as a potential conflict of interest.
