# Supplementary material for: Unusual Cysteine Content in V1 Region of gp120 From an Elite Suppressor That Produces Broadly Neutralizing Antibodies
Source: Front Immunol. 2019 May 15;10:1021. doi: 10.3389/fimmu.2019.01021 (PMC6530427; doi:10.3389/fimmu.2019.01021)
Supplement: Supplementary file 1 [file Table_1.docx]

**Supplementary Table 1. Screening of 25 Elite Suppressors and 4 Normal Progressors for Neutralization Breadth.**

Patient samples exhibiting control of virus replication without anti-retroviral therapy were obtained from the SCOPE cohort (University of California, San Francisco) and were screened using Monogram Biosciences’ PhenoSense® neutralization assay for breadth against a panel of 22 internationally recognized viruses. Each assay included acceptability criteria to ensure that interassay variation between IC50s, measured with reference standards, fell within 2.5-fold 95% of the time. The neutralizing antibody titer (IC50) is defined as the reciprocal of the plasma dilution that produces a 50% inhibition in target cell infection. Values in grey represent neutralization titers that are at least three times greater than those observed against the negative control (aMLV). The clade B NL4-3 and JRCSF viruses were included as CXCR4- and CCR5-dependent positive controls, respectively. Z23 is a reference serum possessing broadly neutralizing antibodies.
