# Supplementary material for: Unusual Cysteine Content in V1 Region of gp120 From an Elite Suppressor That Produces Broadly Neutralizing Antibodies
Source: Front Immunol. 2019 May 15;10:1021. doi: 10.3389/fimmu.2019.01021 (PMC6530427; doi:10.3389/fimmu.2019.01021)
Supplement: Supplementary file 2 [file Data_Sheet_1.PDF]

	Virus	Clade	ID50 (1/dilution)																													
			Elite Suppressors																								Normal Progressors				Neg. Control	Pos. Control
			ES01	ES02	ES03	ES04	ES05	ES06	EN2	ES08	EN3	ES10	ES11	ES12	ES13	ES14	ES15	ES16	ES17	ES18	ES19	ES20	ES21	ES22	ES23	ES24	ES25	NP01	NP02	NP03	NP04	TB	Z23
Simek Panel	94UG103	A	<20	<20	26	22	<20	29	1,292	41	302	81	23	41	23	67	<20	111	<20	45	23	64	32	<20	30	60	92	82	99	41	41	22	222
	92BR020	B	144	106	<20	<20	<20	61	1,282	216	249	46	21	208	72	590	152	425	47	221	151	122	133	<20	251	<20	123	388	170	78	179	<20	347
	93IN905	C	55	41	<20	<20	<20	73	227	288	81	113	26	111	87	253	93	147	44	78	102	113	108	<20	82	136	421	299	98	136	59	<20	388
	M-C-026	C	30	<20	<20	26	<20	24	34	39	27	63	32	50	<20	68	<20	63	21	68	53	58	<20	<20	27	127	573	444	154	100	62	<20	242
	92TH021	AE	25	26	<20	24	<20	23	37	77	65	86	32	43	39	106	74	51	<20	43	30	55	88	<20	44	38	148	76	34	71	32	<20	230
	M-A-002	A	46	21	<20	<20	<20	<20	25	26	92	51	<20	22	<20	29	<20	22	<20	27	<20	41	25	<20	<20	26	86	75	30	34	<20	27	<100
	M-A-006	A	21	21	<20	23	<20	27	92	32	505	36	25	25	<20	42	23	49	<20	65	55	78	32	<20	39	52	54	406	48	38	36	<20	329
	SF162	B	438	20,489	25	29	123	6,290	16,506	37,146	541	3,484	136	21,922	3,468	54,261	12,511	8,361	6,499	8,622	966	4,189	5,885	<20	2,937	743	12,564	22,893	699	41,029	9,408	<20	24,534
	1196	B	84	100	<20	<20	<20	59	5,410	132	131	144	34	203	87	500	125	218	49	66	33	144	90	<20	52	24	182	230	582	255	207	<20	487
	TRO	B	22	25	<20	<20	<20	54	707	67	1,011	209	33	29	29	188	106	448	<20	616	<20	76	<20	<20	21	36	257	64	195	56	89	25	488
	JRFL	B	24	24	<20	20	<20	28	2,333	<20	178	91	31	48	80	108	66	67	<20	39	<20	79	29	<20	<20	<20	56	35	435	64	127	<20	661
	BG1168	B	<20	<20	<20	<20	<20	24	70	<20	45	80	39	20	<20	36	41	30	<20	96	<20	75	<20	<20	27	<20	64	78	120	46	60	<20	163
	QHO692	B	28	23	<20	<20	<20	37	448	<20	71	127	31	29	24	144	40	76	<20	<20	<20	34	<20	<20	<20	<20	77	53	108	34	45	<20	187
	REJO	B	33	32	<20	<20	<20	51	171	110	178	87	28	53	30	177	133	129	23	39	<20	53	32	<20	37	32	246	62	141	59	101	<20	607
	M-SC-B-006	B	<20	<20	<20	<20	<20	<20	96	<20	164	66	36	28	<20	<20	<20	81	<20	76	<20	75	<20	<20	<20	27	20	57	140	73	31	32	153
	APV-16	B	39	21	<20	<20	<20	43	92	45	443	98	31	32	23	135	40	64	<20	<20	<20	51	70	<20	34	80	159	65	135	90	77	<20	826
	M-Chr-B-013	B	26	<20	21	23	<20	22	44	21	123	84	38	34	<20	22	<20	50	<20	21	<20	31	<20	<20	21	29	<20	64	112	54	51	31	297
	PVO	B	<20	<20	<20	<20	<20	<20	2,875	33	403	66	30	23	<20	22	20	46	<20	<20	<20	43	<20	<20	40	<20	42	47	88	64	29	<20	456
	M-C-003	C	38	<20	<20	<20	<20	<20	<20	26	45	29	<20	<20	<20	35	20	22	<20	21	<20	29	27	<20	<20	<20	<20	32	40	<20	22	<20	<100
	M-C-020	C	22	34	26	32	<20	51	2,398	46	22	73	35	56	24	52	33	52	<20	26	31	40	35	<20	33	<20	46	75	25	77	42	23	<100
	M-D-006	D	<20	<20	<20	<20	<20	<20	793	<20	282	51	<20	<20	<20	35	<20	73	<20	<20	<20	28	<20	<20	<20	<20	35	41	<20	28	25	<20	240
	M-D-009	D	144	107	<20	<20	<20	40	664	175	170	286	22	59	117	372	174	195	49	86	145	115	137	<20	49	76	178	157	106	144	202	<20	250
Controls	aMLV (neg.)	N/A	<20	<20	<20	<20	<20	<20	<20	<20	<20	23	<20	<20	<20	<20	<20	24	<20	<20	<20	20	<20	<20	<20	<20	<20	<20	<20	<20	<20	<20	<100
	JRCSF (pos.)	B	<20	68	<20	24	<20	33	3,149	71	556	123	28	111	29	161	79	229	<20	65	39	110	115	<20	67	76	165	260	344	82	46	<20	610
	NL43 (pos.)	C	1,722	1,354	27	<20	47	856	2,109	3,305	9,908	231	48	1,269	1,764	5,241	2,650	1,114	1,057	2,485	2,195	1,249	1,368	<20	722	1,164	611	2,081	2,509	3,465	1,678	<20	4,795
